# Combined *in vivo* and *silico* assessment of melatonin’s protective effects on rifampicin-induced liver damage in rats

**DOI:** 10.1038/s41598-025-16453-z

**Published:** 2025-08-24

**Authors:** Khaled Abo-EL-Sooud, Hagar Hesham, Maryam Saeed, Sarah Mohamed, Fady Sayed Youssef

**Affiliations:** Department of Pharmacology, Faculty of Veterinary Medicine, Cairo University, Giza, Egypt

**Keywords:** Antioxidant, Cinnamic acid, Docking, Hepatotoxicity, Melatonin, Rifampicin, Diseases, Health care

## Abstract

Liver injury is a globally serious problem that may be observed post-chemotherapeutic administration in chronic crises such as tuberculosis (TB). Rifamycin (RIF), particularly, is an indispensable treatment regimen for TB with a significant negative hepatic impact. Therefore, this research aims to assess the restorative role of melatonin (MEL) against rifampicin (RIF)-associated hepatic damage in rats. Moreover, to investigate the ultimate mechanism of the antioxidant potential of MEL with multimodal assessment and in silico molecular conformation. Adult male *Wistar* albino rats were weighed and divided into four groups of ten rats each. The control group received the vehicle (0.5 ml/day), the RIF-intoxicated group (100 mg/kg/day orally), the MEL-treated group (10 mg/kg/day intraperitoneally), and the Co-administered RIF and MEL group at the same regimen for 21 consecutive days. Blood and hepatic tissue samples were obtained for biochemical, histological, and molecular studies. High in vitro antioxidant scavenging potential of MEL with an IC_50_ of 94.66 µg/ml was attained using a 2,2-diphenyl-1-picrylhydrazyl (DPPH) assay. Fourier transform infrared spectroscopy (FTIR) and gas chromatography-mass spectrometry (GC–MS) analyses of MEL indicated the presence of mainly cinnamic acid, m-(trimethyl-silyl ester), in addition to several reactive antioxidant moieties. The plasma levels of aspartate aminotransferase (AST), alanine aminotransferase (ALT), alkaline phosphatase (ALP), and lipid profiles were significantly reduced in the MEL + RIF co-administered group in contrast to the RIF-treated group (*P < 0.05*). Moreover, MEL significantly decreased the hepatic oxidative biomarkers malondialdehyde (MDA) and significantly increased the estimated antioxidant enzyme levels (*P < 0.05*) of superoxide dismutase (SOD) and glutathione peroxidase (GPX). Histological evaluations revealed mild hepatic injuries and inflammatory cellular infiltration in MEL-treated animals. Molecular docking explored the high-affinity interaction of MEL and cinnamic acids with hepatic cytochrome P450 isoform 3A4 (CYP3A4) protein target, confirming their hepatic restorative potential. This study endorses the antioxidant potential role of MEL in RIF-hepatic injury.

**Supplementary Information:**

The online version contains supplementary material available at 10.1038/s41598-025-16453-z.

## Introduction

Liver injury is commonly associated with extensive drug administration, especially in chronic diseases like TB, and the rate of liver injury is annually increased with antitubercular agents^1^. This injury is closely linked with oxidative damage, cellular degeneration, and inflammatory reactions^2^.

TB is still a major global health concern, and the annual deaths were approximately 1.5 million^3^. RIF is an eminent anti-tuberculosis drug and is extensively utilized to treat pulmonary TB alone and in a combined therapy approach with isoniazid, as stated by the World Health Organization^4^. Recently, in silico docking suggested that RIF has a favorable binding affinity with the COVID-19 protease enzyme, recommending its therapeutic and prophylactic roles^5^. The combined regimen for adult respiratory TB treatment comprises two months of treatment with isoniazid and RIF. The hepatotoxicity incidence of drug-induced liver injury has been increased with that combination, especially in cirrhotic patients^6^. The metabolism of isoniazid is increased with RIF as it is a CYP3A4 inducer and hastens isonicotinic acid and hydrazine (toxic metabolites) by stimulating isoniazid hydrolases. Finally, hydrazine is further metabolized to N-hydroxy acetyl hydrazine with potential toxicity^7^. Consequently, RIF augments the hepatotoxic activity of other anti-tuberculosis drugs.

Prolonged RIF administration would substantially raise the hazard of liver damage through multiple pathways^8^. Hepatotoxic damage induced by RIF is a significant clinical challenge^9^.

MEL is a pleiotropic molecule with functional diversity. The indirect antioxidant and direct radical scavenging action of indole has been proven in earlier literature. It has a potent efficacy to protect against detrimental side effects, as well as the toxicity of a broad variety of chemotherapeutic agents^10^. The endogenous concentration of MEL differs extensively from lower blood levels to higher intracellular distribution, specifically in the mitochondria, which supports its capability to overcome oxidative damage and apoptosis. With the increased incidence of TB, particularly in low-income countries, and the essential use of RIF, with its undesirable effects, the use of adjunctive therapy with a supportive agent like MEL becomes clinically urgent. MEL has potent antioxidant and immunomodulatory properties that could potentially mitigate the side effects of RIF and enhance immune recovery associated with TB.

The high safety and endogenous existence of MEL make its use as an adjunct therapy with the expected toxic remedies look imperative and valuable benefits, especially in immunosuppressive chronic diseases such as TB^10^. A reasonable dosage of 10 mg/kg of MEL proved to be a safer and more efficient mechanism for reducing non-alcoholic fatty liver disease induced by a high-carbohydrate, high-fat diet in rats^11^.

MEL is a potent free radical scavenger against reactive oxygen species (ROS) and reduces oxidative damage to lipids and nucleoproteins wherever oxidative toxic metabolites are produced^12^. This might be because of MEL’s chemical structure and how it interacts with reactive species, which makes it a far more effective scavenger with a far larger relevance and protective value against oxidative damage^13^. The receptor-independent activity refers to MEL’s capability to detoxify free radicals, hence defending vital structures damaged by oxidative stress, radiation exposure, and drug toxicity^14^. This function of MEL proposes its action as a signaling moiety in the interrelation of MEL with diverse signaling structures as mitogen-activated protein kinase (MAPK) cascades, Ca^2+^ influx, hydrogen peroxide (H_2_O_2_), and nitric oxide (NO)^15^. Moreover, MEL also reportedly chelates transition metals, so it lessens the production of the extremely toxic hydroxyl radical, leading to the alleviation of oxidative distress^16^. As RIF activates CYP3A4 enzyme-induced toxic drug metabolites by pregnane X receptor (PXR), therefore, our hypothesis is to elucidate if MEL exerts its antioxidant protective effect through the down-regulation of PXR and CYP3A. To date, there is no data assessing the antioxidant activity of MEL resolving RIF-initiated hepatotoxicity in rats. Hence, in this research, we inquired about the potential protective impact of MEL to overcome oxidative hepatic damage in rats caused by RIF with multimodal assessment and in silico molecular conformation.

## Results

### GC-MS–MS, besides FTIR analyses

**Fig. 1:**
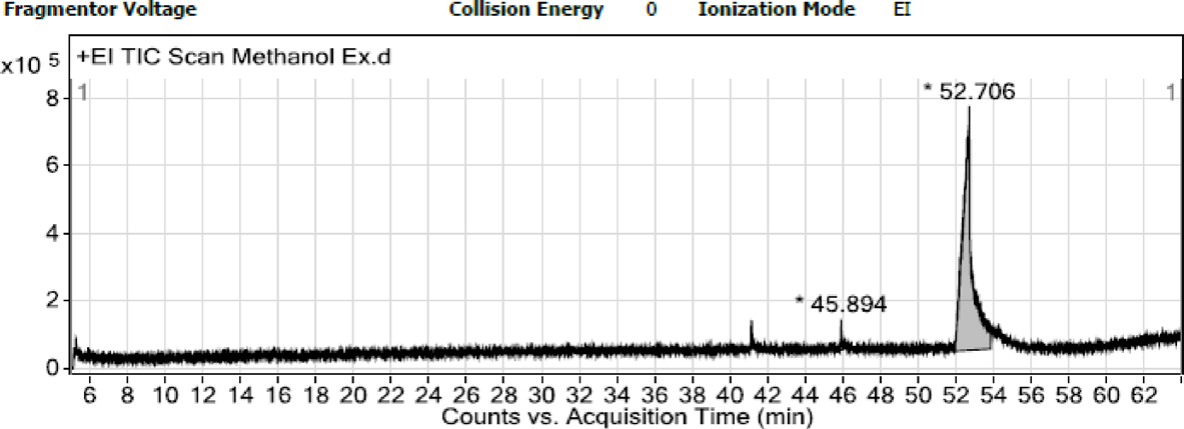
GC-MS chromatogram of melatonin.

**Fig. 2:**
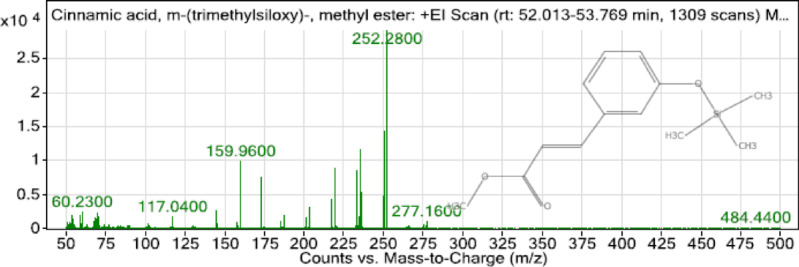
MS of the peak with the retention time of 52.706 min from electron ionization mass spectra of library research results (cinnamic acid).

GC–MS analysis was conducted, and one main compound was identified as cinnamic acid, m-(trimethyl-silyl ester) (Fig. 1). The MS of the cinnamic acid is illustrated in Fig. 2.

**Fig. 3:**
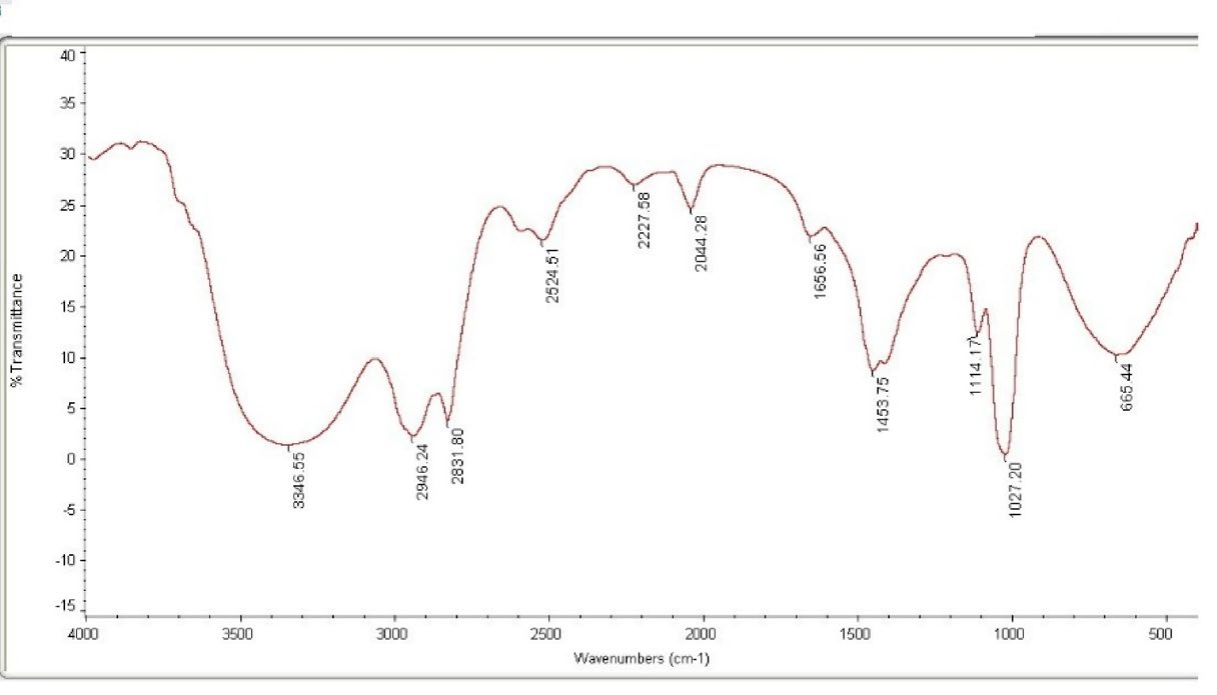
FTIR spectra of melatonin.

The FTIR spectra generally showed characteristic absorption peaks representing a mixture of several reactive moieties (Fig. 3). The range of 1027–1114 cm^−1^ wavenumbers represents the C-N amine group and S = O sulfate moiety, while the peak at 1453 cm^−1^ represents the C-H methyl group. The band at 1656 cm^−1^ is probably from twisting C–C stretch vibrations, as well as the existence of aromatic moieties. The range of 2044–2946 cm^−1^ wavenumbers is, in general, allocated to the S-H thiol bond attached to the N = C = O isocyanate group. We observed one large band at 3346 cm^−1^ in the FTIR spectra of MEL and assigned it to the stretching vibrations of the N-H amide (indole) group. The indole moiety of MEL’s structure plays a central role in free radical scavenging due to its electron-rich aromatic system. This makes indole-containing compounds inherently effective at neutralizing oxidative stress.

Momentarily, to summarize the interpretation of FTIR spectra, the vibrations can be separated into three assemblies: the heterocyclic indole ring, the ethylamine side chain (sulfate group), and a methoxy group, OCH_3_. Moreover, sulfated derivatives of MEL exhibit increased hydrophilicity and may act as storage or transport forms, modulating MEL’s systemic antioxidant action. Together, these functional groups could serve as optimized antioxidants in oxidative stress-amended TB infection or during the RIF regimen.

#### In vitro antioxidant activity

A significant in vitro DPPH scavenging action consistent with high antioxidant potential was shown by the methanolic extract of MEL with an IC_50_ of 94.66 µg/ml. The ability of gradual concentrations of MEL to scavenge free radicals is dose-dependent figure with antioxidant activity.

### Effects on the body weight and hepatosomatic index of rats

The outcome of orally administered RIF on the ultimate body weight or body weight gain, contrary to the control group of rats, is shown in Table 1. No mortality was recorded in any of the tested rats. The control group had a mean body weight change of 67.40 g. The RIF-intoxicated group showed a significant decrease of 59% and 23% alone and with MEL, respectively. It was observed that food and water intake were decreased in RIF-treated rats. A significant (*P < 0.05*) rise of 120% was exhibited in the MEL-treated group compared to the control group (Table 1). Eventually, the mean liver weight was 9.8 g for the normal rats. The weight of the liver rose by about 15% in the Mel-treated group, while RIF significantly lessened the hepatosomatic index in contrast to the control group (*P < 0.05*), as displayed in Table 1.

**Table 1:** Effect of MEL on body and liver weight change, hepatosomatic index of Wistar male rats exposed to oral dosing of RIF for 21 days.

Estimated parameter	Control	RIF	MEL	RIF + MEL
Initial body weight (g)	153.40 ± 8.01	142.60 ± 8.79	152.10 ± 1.65	145.33 ± 3.90
Final body weight (g)	220.80^a^ ± 7.55	170.20^c^ ± 8.20	237.00^a^ ± 6.67	197.25^b^ ± 5.65
Body weight change (g)	67.40^a^ ± 3.08	27.60^d^ ± 4.36	84.90^b^ ± 4.17	51.92^c^ ± 3.38
%	43.94^a^ ± 1.11	19.35^c^ ± 0.95	55.82^b^ ± 1.28	35.73^a^ ± 1.07
Liver weight (g)	7.67^a^ ± 0.13	5.50^b^ ± 0.15	9.40^c^ ± 0.10	7.80^a^ ± 0.14
Hepatosomatic index (%)	3.47^a^ ± 0.05	3.23^b^ ± 0.03	3.97^c^ ± 0.4	3.95^c^ ± 0.02

Hepatosomatic index was calculated as the ratio between liver weight and body weight in grams ×100.

The values shown are means ± SE. (*n* = 10). Values in the same row carrying different superscripts are significantly different at *P* < 0.05.

### Effects on hepatic function indicators

The plasma hepatic enzymes (AST, ALT, and ALP) were significantly (*P* < 0.05) elevated, while the albumin concentration was significantly decreased following daily exposure to RIF for 21 days compared to the control group. In contrast, MEL significantly (*P* < 0.05) decreased the activities of these enzymes by 28.71%, 34.56%, and 29.82% and significantly increased albumin concentration by 17.66% in the RIF-treated group (Table 2).

**Table 2:** Effect of MEL on biochemical parameters and lipid profiles in Wistar male rats exposed to oral dosing of RIF for 21 days.

Estimated parameter	Control	RIF	MEL	RIF + MEL
**Albumin (g/dl)**	3.90^a^ ± 0.11	2.83^d^ ± 0.15	3.55^c^ ± 0.08	3.33^b^ ± 0.078
**AST (U/l)**	43.00^a^ ± 2.52	101.19^c^ ± 2.08	45.33^a^ ± 1.47	72.33^b^ ± 1.27
**ALT (U/l)**	32.67^a^ ± 2.40	81.33^b^ ± 1.76	34.33^a^ ± 1.20	53.00^a^ ± 1.73
**ALP (IU/l)**	53.33^a^ ± 2.96	114.33^d^ ± 2.60	55.67^a^ ± 1.41	80.00^b^ ± 1.53
**Total cholesterol (mg/dl)**	47.33^a^ ± 1.76	93.33^c^ ± 2.73	49.67^a^ ± 1.76	58.33^a^ ± 1.20
**Triglyceride (mg/dl)**	52.00^a^ ± 1.72	100.00^c^ ± 2.65	53.00^a^ ± 1.53	69.00^b^ ± 1.15
**HDL (mg/dl)**	38.31^a^ ± 1.09	24.21^c^ ± 1.21	40.37^a^ ± 0.88	31.73^b^ ± 0.95
**LDL (mg/dl)**	16.56^a^ ± 1.00	47.00^c^ ± 1.50	17.08^a^ ± 1.03	25.54^a^ ± 0.72
**Cholesterol: HDL**	1.24^a^ ± 0.09	3.86^c^ ± 0.13	1.18^a^ ± 0.07	1.83^b^ ± 0.06

AST: Aspartate aminotransferase; ALT: Alanine aminotransferase; ALP: alkaline phosphatase; LDL: low-density lipoprotein; HD: High-density lipoprotein. The values shown are means ± SE. *n* = 10. Values in the same row carrying different superscripts are significantly different at *P* < 0.05.

### Effects on lipid profile

Variations in the serum lipid profile of rats orally exposed to RIF for 21 days and those treated with MEL injection are shown in Table 2. In comparison with the control group, the serum levels of total cholesterol (TC), triglycerides (TG), low–density lipoprotein cholesterol (LDL), and cholesterol: high–density lipoprotein cholesterol (HDL) ratio were significantly (*P* < 0.05) increased in RIF-exposed rats. Contrarily, a significant drop in the HDL values was obvious in RIF-treated rats relative to the control rats. MEL significantly improved the lipid profiles and cholesterol: HDL ratio when it was administered concurrently with RIF, almost proximate to the control non-treated rats (Table 2).

### Effects on hepatic oxidative stress and lipid peroxidation indicators

**Fig. 4:**
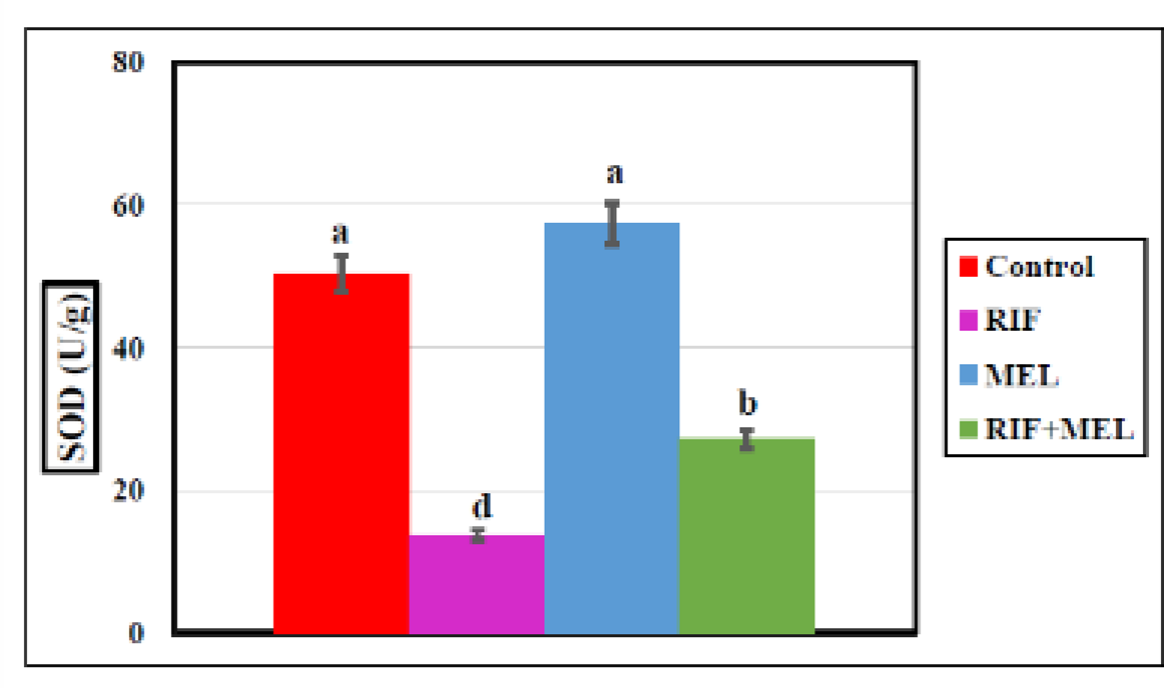
Effect of melatonin on hepatic SOD (antioxidant enzyme) in Wistar male rats exposed to oral dosing of rifampicin for 21 days. Columns carrying different superscripts are significantly different (one-way ANOVA) (*P < 0.05*).

**Fig. 5:**
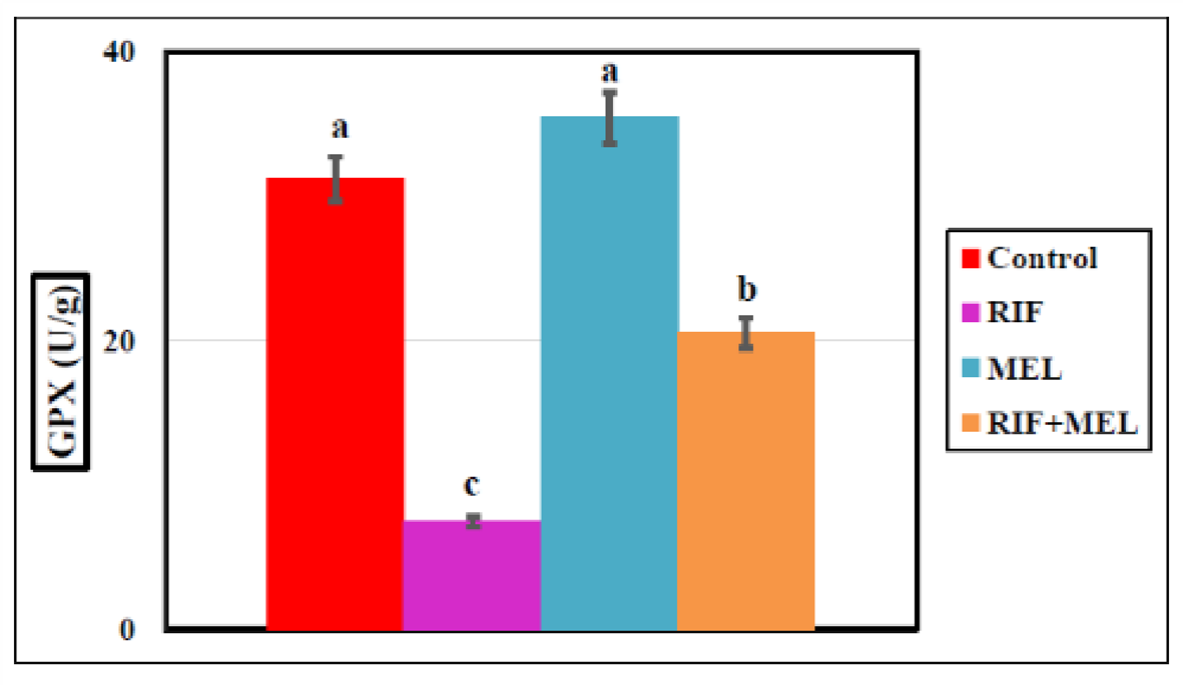
Effect of melatonin on hepatic GPX indicator in Wistar male rats exposed to oral dosing of rifampicin for 21 days. Columns carrying different superscripts are significantly different (one-way ANOVA) (*P < 0.05*).

**Fig. 6:**
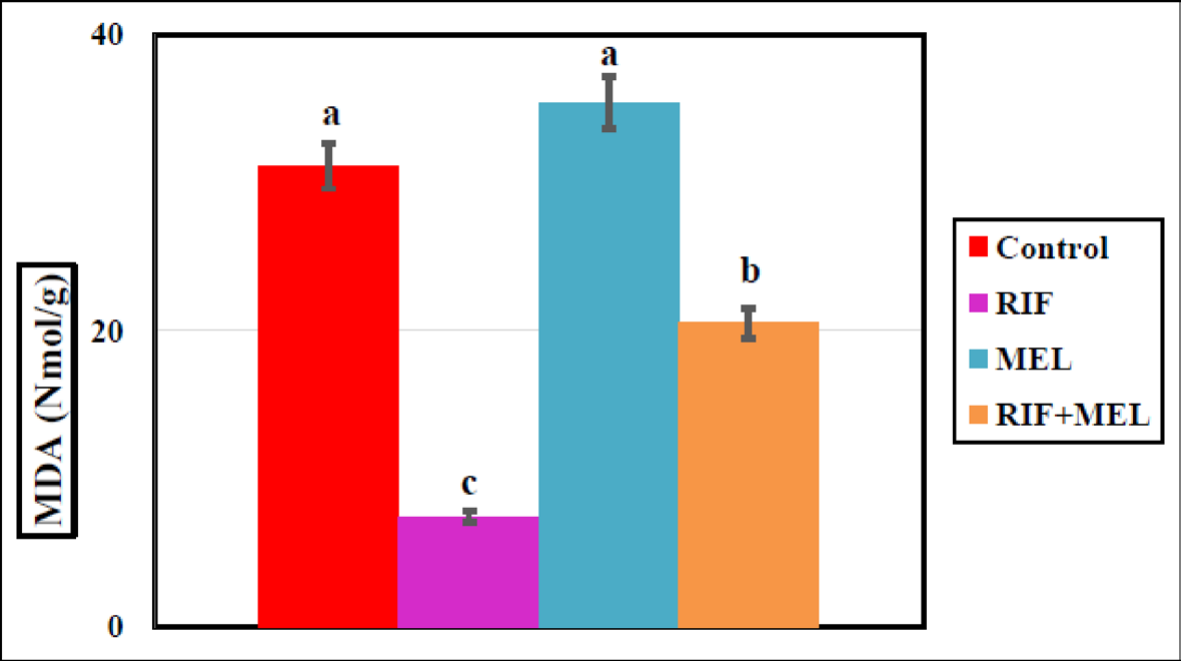
Effect of melatonin on hepatic MDA (lipid peroxidation indicator) in Wistar male rats exposed to oral dosing of rifampicin for 21 days. Columns carrying different superscripts are significantly different (one-way ANOVA) (*P* < 0.05).

The homogenate’s SOD, GPX, and MDA levels in hepatic tissue, RIF-treated rats exhibited a marked reduction in SOD and GPX concentrations by 72.6% and 76%, respectively, but the MDA concentration increased (79%) substantially compared to the normal group, indicating that RIF exaggerated the hepatic oxidative stress. MEL induced significant rises in SOD and GPX and markedly decreased the MDA level (*p* < 0.05) in the RIF-treated groups (Figs. 4, 5 and 6), indicating that MEL improved hepatic antioxidant activity. The data obtained were provided in Supplementary Table 1.

### Histological results

**Fig. 7:**
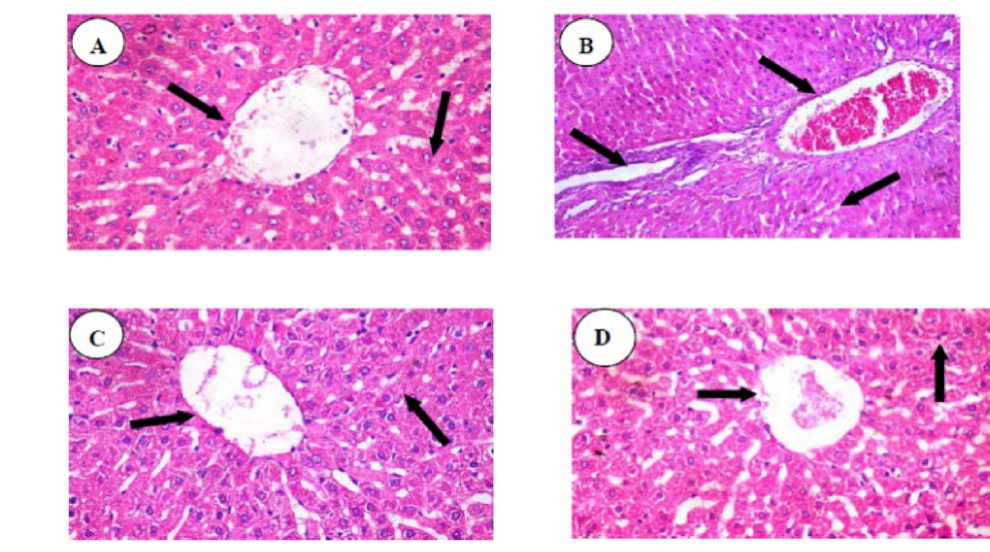
Histological examination of liver stained with H&E×400. **(A)**: Control normal liver showed a normal histological picture with normal hepatocytes and portal areas (arrowhead). (**B)**: RIF-treated liver showed dilated and congested hepatoportal blood vessels and the hyperplastic bile duct (arrowhead). A diffuse vacuolar degeneration in the hepatocytes with ring appearance nuclei was observed (arrowhead) **(C)**: MEL revealed normal hepatic parenchyma, normal blood sinusoids, and central vein (arrowhead) **(D)**: Co-treatment of RIF with the MEL-treated group exhibited a significant improvement in its histological picture except for paracentral small focal areas of enlarged hepatocytes with faint cytoplasm (arrowhead).

The control group represented normal hepatocellular architecture without any sign of necrosis, along with a well-preserved hepato-lobular pattern and normal size of central vein and blood sinusoids [Fig. 7: (A)]. These findings were also very similar in the MEL-treated group [Fig. 7: (C)]. The severity of the hepatic changes was more obvious in the RIF group, as dilated and congested hepatoportal blood vessels and hyperplastic bile ducts were observed. A diffuse vacuolar degeneration in the hepatocytes was observed in lobular inflammation with congestion and dilatation of the central and portal veins, and blood sinusoids. Thickening of the wall of blood vessels with vasculitis, as well as perivascular edema, was noted. [Fig. 7: (B)]. Co-treatment of the RIF and MEL-treated group exhibited prominent improvement in its histological picture, except for mild congestion in a few sections, paracentral small focal areas of enlarged hepatocytes with faint cytoplasm, and dilated central vein [Fig. 7: (D)]. Moreover, the hepatic tissue scoring confirmed the histopathological findings recorded in Table 3.

**Table 3:** Effect of MEL on histopathological scoring (0–5) in the liver of Wistar male rats exposed to oral dosing of RIF for 21 days.

Parameter	Control	RIF	MEL	RIF + MEL
**Cellular necrosis**	0	5	0	0
**Hepatocyte degeneration (vacuoles)**	0	5	0	1
**Portal vein disruption**	0	4	0	1
**Bile duct hyperplasia**	0	5	0	0
**Congestion in hepatoportal blood vessels**	0	5	0	1

The scoring was made on a six-point scale according to Maiti et al.^26^.

### Molecular Docking analysis

**Fig. 8:**
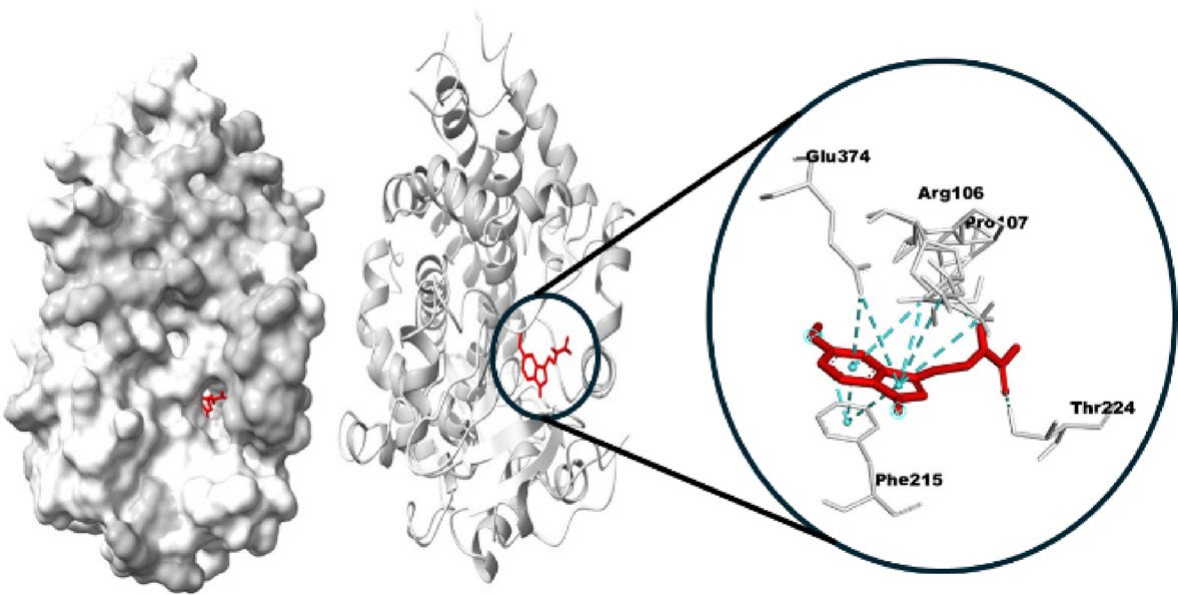
Docked 3D structure interaction between the ligands of melatonin with the liver corrective chain A of the CYP3A4 enzyme target.

**Fig. 9:**
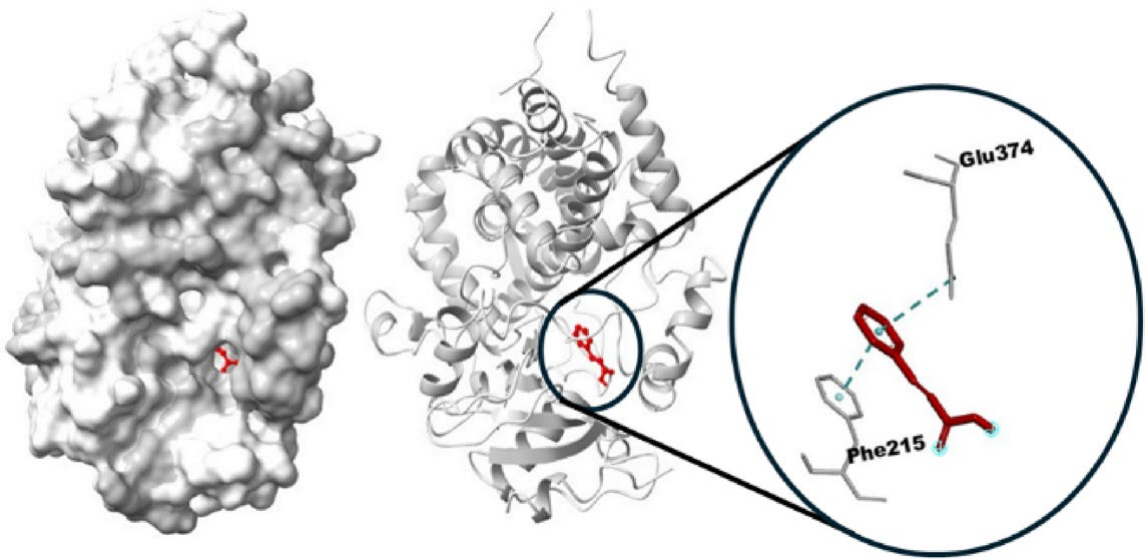
Docked 3D structure interaction of cinnamic acid with CYP3A4 complex.

**Fig. 10:**
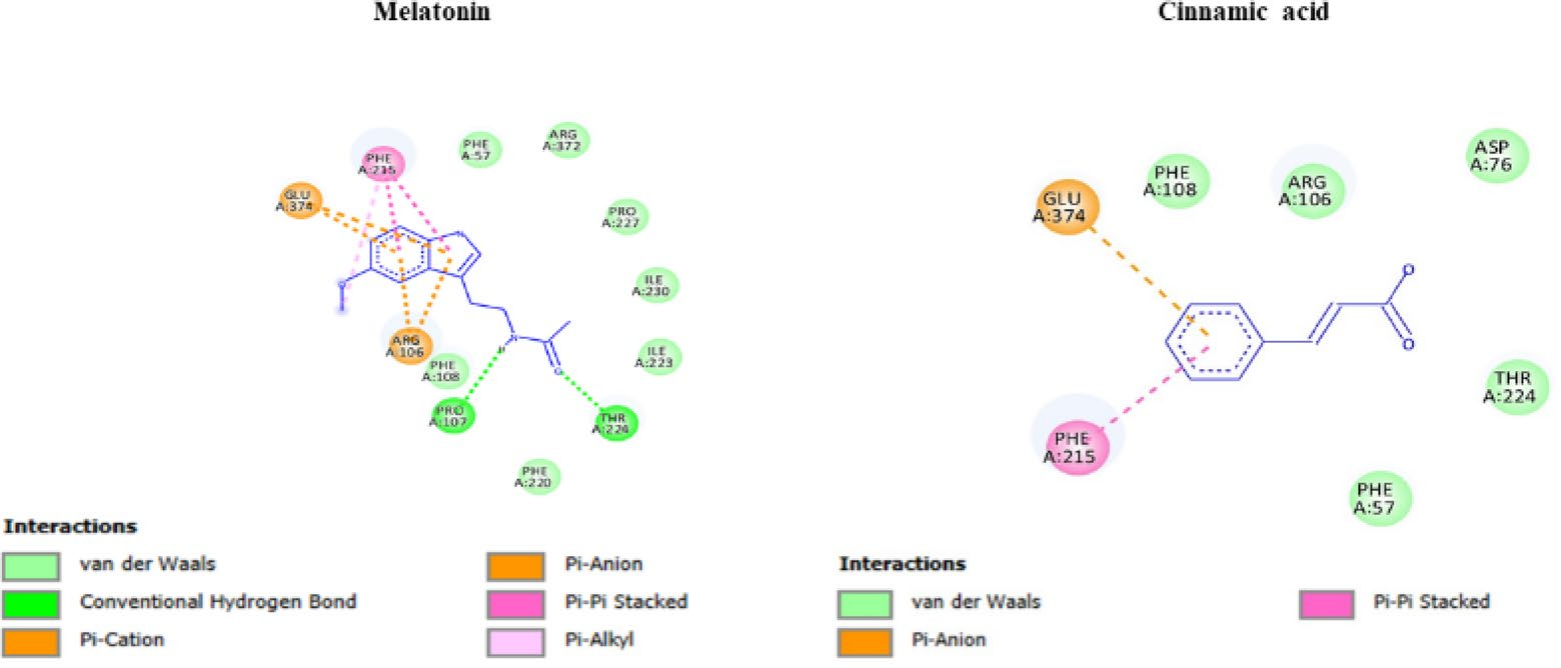
2D-Molecular docking of the interaction between CYP3A4 and the ligands with different bonds.

The in-silico investigations revealed a high binding affinity between MEL and CYP3A4, which is to be considered as a potential mechanism. The estimated Gibbs free energy equals − 8.8 kcal/mol, indicating a high binding affinity by two conventional hydrogen bonds between MEL and chain A of CYP3A4. In addition, MEL can further bind to CYP3A4 with six potential electrostatic Pi bonds, specifically indicating a favorable and stable interaction. This high affinity is primarily attributed to the formation of two conventional hydrogen bonds between MEL and chain A of CYP3A4, supporting the metabolic engagement at the enzymatic active site. The findings support the hypothesis that this interaction could have important implications for MEL’s bioavailability and antioxidant efficacy in TB patients undergoing Rifamycin-based treatment (Fig. 8). CYP3A4 was further found to interact with cinnamic acid by an electrostatic bond with CYP3A4 (7.3 kcal/mol) (Fig. 9). Moreover, the hydrophobic interactions with MEL are clarified in the 2D diagram (Fig. 10). The interactions play a significant role in stabilizing the ligand-protein complex, as depicted in the 2D interaction diagram. Specifically, a bonding interaction is observed between the cinnamic acid moiety and glutamate residue of CYP3A4, which may contribute to electrostatic stabilization at the binding interface. These interactions with cinnamic acid highlight the importance of both polar and non-polar contacts in governing the binding affinity of the ligand to CYP3A4 (Fig. 10).

## Discussion

The World Health Organization (WHO) has expressed worry about the increased TB incidence in many developing countries that may be attributed to immunodeficiency diseases, malnutrition, and population-attributable factors^17^. RIF is an effective remedy for TB, although it has been known as its key promoter for severe hepatotoxicity after long-term administration^18^.

Nowadays, researchers are fascinated by the mechanisms of MEL in oxidative stress, lipid peroxidation, and its talent pharmacological benefits. Therefore, researchers focused their efforts on the protective role of MEL on liver injuries from diverse sources.

GC–MS analysis of MEL demonstrated the existence of cinnamic acid, m-(trimethyl-silyl ester) as the main compound. Now, MEL is documented as a potent scavenger against reactive oxygen species (ROS) and nitrogen species^19^, especially those formed from peroxynitrite, and blocking transcriptional factors, including proinflammatory cytokines, or by influencing the antioxidant enzymes’ expression^20^ at mmol levels.

Consequently, it is not amazing that several MEL substituents have been established, synthesized, and evaluated, leading to new multitarget-directed ligands (MTDL). The combination of MEL with cinnamic acid exhibited a potential synergistic antioxidant outcome for drug toxicities^21^. In the mission for new MTDL moieties, nine new cinnamate-MEL hybrids were envisaged as multitarget nuclear factor erythroid-2 related factor (Nrf2) inducers and scavengers^22,23^. The FTIR spectra of MEL generally showed a mixture of several reactive moieties. Confirmedly, Florio et al.^24^ stated that MEL possessed a single methyl amide in addition to the side chains, including the N-acetyl ethylamine and methoxy group positioned at 3 and 5 positions.

The high antioxidant potential in vitro efficacy of MEL, with an IC_50_ of 94.66 µg/ml, was very close to the value recorded by Asad-Ullah et al.^25^.

Our results revealed that exposure to RIF significantly reduced the ultimate body weight or body weight gain. Maiti et al.^26^ confirmed the same results as RIF-induced significant alterations in the relative body and liver weights, which may be due to a decrease in food and water intake in RIF-treated rats. Nonetheless, MEL caused a significant rise in body weight gain and hepatosomatic index alone and with RIF.

The liver weight had increased in the control group and the MEL-treated group, along with body weight. While the RIF-treated rats showed a significant decrease in liver weight (hepatosomatic index), this might be attributed to the accumulation of lipids^26^. Supportive findings were obtained as the values of TC, TG, LDL, and cholesterol: HDL ratio were significantly (*P* < 0.05) elevated in RIF-exposed rats.

Consistent with these results, RIF-induced mixed hepatotoxicity *via* cholestasis and hepatocellular injury^27^. In the current analysis, the RIF-induced liver injury was assessed by estimating blood transaminases, ALP, albumin, lipid profiles, hepatic antioxidant markers, and histological alterations staining according to appropriate studies^2^. MEL could reduce hepatic oxidative damage by potentiating mitochondrial functions, protecting the hepatic architecture from apoptosis and necrosis^12^.

The toxicity-related hepatic markers like ALT, AST, and ALP increased significantly in the blood of RIF-treated rats. The leakage of the intracellular enzymes occurred due to oxidative stress-induced LPO-mediated membrane damage^27,28^.

RIF induced a significant decrease in the concentration of plasma albumin; this figure is usually associated with hepatic damage, as the hepatocyte is where plasma protein synthesis is utmost. It induces inflammatory mediators; RIF also augments cytokine-induced NO release and interleukin 8 (IL-8) in hepatocytes. The high NO release subsequently disrupts the triggering of other immunomodulators, which explains the massive influence of RIF on immunity^29^. Hepatotoxicity is a consequence of the release of pro-inflammatory cytokines such as TNF-α, interleukins, and ROS accumulation. The decline in blood protein is accompanied by a reduction in DNA and RNA hepatic contents by base-pairing alterations induced by ROS cellular damage^30^. In diverse means, RIF triggers endoplasmic reticulum stress, which results in cholestasis, in addition to hepatic oxidative damage, bile acid accumulation, and CYP3A4 enzyme-induced toxic drug metabolites by pregnane X receptor (PXR). Although the organism’s short-term stress reaction aids in its resistance to toxins, a prolonged stress response exacerbates liver damage. Consequently, endoplasmic reticulum stress may be associated with the apoptotic damage and the “adaptable” nature of RIF^9^.

Chawla et al.^31^ reported that MEL (10 mg/kg/day) had hepatoprotective efficacy as compared to RIF-intoxicated rats; the integrated treatment with MEL hindered the decline in weights of both body and liver, and it also hindered liver enzyme activity and liver protein levels.

Our investigations indicated that the blood concentrations of TC, TG, LDL, and the cholesterol: HDL ratio were significantly (*P* < 0.05) elevated in RIF-exposed rats, with a significant decline in the HDL level relative to the control rats. In this respect, Huang et al.^32^ stated that hepatic TG was considerably elevated in RIF-treated mice. Moreover, Ahmed et al.^33^ stated that post-RIF therapy in pulmonary TB, the levels of cholesterol, HDL, and LDL significantly increased.

Pre-treatment with MEL in hepatotoxic rats normalized the biochemical plasma concentrations of ALT, AST, ALP, total bilirubin, total lipids, TC, TG, and total proteins and upgraded the hepatic level of the oxidative stress parameters of MDA, GPX, SOD, reduced glutathione (GSH), and catalase (CAT). Moreover, it greatly improved the histological alterations^34^. Moreover, MEL may alleviate oxidative stress also by stimulating some antioxidative enzymes, i.e., SOD^35^, and GPX^36^.

MEL readily penetrates cellular membranes owing to its size and lipophilicity. Additionally, MEL has been proven to be an effective DNA protector^37^, proteins, as well as lipids in cell membranes^38^. MEL scavenges hydroxyl radicals^39^ and peroxyl radicals^40^, which are produced through unsaturated fats, resulting in the initiation of lipid peroxidation.

The antioxidant radical scavenging properties of MEL and its metabolites occur chiefly through the one-electron transfer process^41^. The first ionization potential (IP) and the electron affinity (EA) are chiefly used to allow measurement of its tendency to give or accept one electron.

RIF induces overexpression of the pregnane X receptor (PXR), a ligand-dependent nuclear receptor transcription factor^42^. Consequently, the CYP3A4 enzyme subset, responsible for the transcriptional function of the ATP-binding cassette ABCB1 transporter^43^. Finally, the lipid buildup in the liver caused by RIF is associated with the upregulation of peroxisome proliferator-activated receptor gamma (PPARγ), which stimulates the expression of perilipin protein that covers the lipid moiety and prevents lipolysis^26^. The molecular hepatotoxicity mechanism of RIF was elucidated by glutathione transferase activity was up-regulated, whereas fatty acid metabolism was down-regulated, causing hepatic necrosis and hyperbilirubinemia. Moreover, the PPARγ signaling pathway, CYP3A4, and glutathione metabolism accelerated dose-dependently in RIF-treated livers^27^.

Moreover, the inducer role of RIF to CYP3A4 for wide chemotherapeutic agents has potential consequences in possible hazardous drug interactions with anticoagulants^44^, and antiretroviral therapy, which was warned in clinical trials of assessment of RIF against SARS-CoV-2 concurrently with further drugs that are also substrates for the hepatic biotransformation of CYP3A4^45^.

RIF is reported as a potent inducer for CYP3A4^46,47^, that triggers oxidative hepatic damage and leads to cytotoxic effects^48,49^. Consequently, intense CYP3A4 induction is considered a serious safety risk factor^50^.

MEL ameliorates RIF-hepatotoxicity due to high affinity with CYP3A4 (Gibbs free energy of −8.8 kcal/mol), which suggests a competitive inhibitory mechanism, associated with the strong induction of cytochrome monooxygenase enzymes. In this respect, Eugenia et al.^50^ emphasized that MEL had a protective antioxidant action against cytochrome spectral activity with high binding affinity to CYP3A4, limiting its catalytic activity.

MEL reduced the expression of mRNA of CYP3A4 through the linking of hydrogen bonds and dynamic alterations in the binding site. Similarly, MEL substantially diminished the hepatic oxidative damage of Oxfendazole, a common benzimidazole member^51^.

Cinnamic acid derivatives were reported to reversely inhibit CYP3A isoforms 3A4, 1A2, 2B, and 2C11, leading to competitive or mixed inhibition^51^.

The hepatoprotective effect of MEL against RIF-induced hepatic damage may be due to disparate mechanisms. Our study suggests a potential mechanism by which MEL ameliorates the strong induction of cytochrome enzymes caused by RIF. MEL can interact with CYP3A4 with hydrogen, electrostatic, and hydrophobic bonds, suggesting a competitive inhibitory mechanism resulting in amelioration of the oxidative stress and subsequent hepatic damage caused by RIF.

Recently, He et al.^52^ evaluated the antioxidant potential of new MEL-C7-cinnamic acid moieties; they stated that the cinnamic acid moiety improves the antioxidant activity of MEL. The exogenous source of MEL significantly increased the accumulation of individual phenolic acids and flavonoids, especially cinnamic acid contents^53^.

Lipopolysaccharide-induced oxidative injury by augmenting cytokines systemic concentrations, and nuclear factor (NF)-κB signaling pathway in hepatocytes^54^. MEL protective activity overcomes LPS‐induced oxidative toxicity by decreasing the expression of mitochondrial NADP oxidase-4 while stimulating Nrf2. Consequently, it is increasing the activity of GPX and cytochrome reductase and inhibiting cell death in LPS‐treated animals^55^.

The in silico docking analysis of MEL with CYP3A4 proposes a mechanistic explanation that aligns with biochemical observations involving RIF-induced toxicity. The strong binding affinity of MEL to CYP3A4 supports the hypothesis that MEL may act as a competitive substrate or modulator of this isoform. This interaction could potentially inhibit excessive CYP3A4-mediated oxidative metabolism triggered by RIF, thereby limiting the generation of ROS and other toxic intermediates. These effects suggest a dual mechanism of MEL: direct radical scavenging and modulation of metabolic enzyme activity, particularly involving the CYP3A4 isoform. The amide group in MEL and the carboxyl group of cinnamic acid contribute to hydrogen bonding and molecular stability, enhancing membrane permeability and potentially establishing electrostatic interactions and a hydrogen bond with the enzyme. This interaction elucidates the importance of both polar and non-polar contacts in governing the binding affinity of the ligand to CYP3A4^51,52^.

The docking data thus provide a molecular framework for understanding how MEL’s interaction with CYP3A4 could contribute to its hepatoprotective and antioxidant effects during RIF therapy, offering strong support for its potential as an adjunctive therapeutic agent.

## Conclusion

Our findings indicated that MEL normalized plasma lipid and oxidative stress markers and lessened the RIF co-hepatotoxic impacts in addition to its potential antioxidant characteristics. Therefore, it is credible to prescribe MEL concurrently as a potential therapeutic regimen for tuberculotic patients for the prevention of RIF-induced hepatotoxicity. Further molecular in vivo studies are warranted to prove the possible explanations from in vitro, in vivo, and in silico studies suggesting that MEL embraces substantial latent hepatoprotective agents by interacting with CYP3A4 pathways involved with consequent oxidative hepatic damage caused by RIF.

## Methods

### Drugs and chemicals

MEL was bought from Sigma-Aldrich (St. Louis, MO, USA). RIF was purchased from Sanofi-Aventis (Barcelona, Spain). Carboxymethyl cellulose (CMC) and Tween 80 were obtained from Advent, India. All of the solutions were freshly prepared daily on each test day. RIF in an aqueous suspension of 0.5% CMC and 0.4% Tween 80 orally (gavage)^56^, whereas MEL was diluted in isotonic saline after dissolving in a minimal amount of ethanol (final ethanol amount is 1%, w/v).

FTIR analysis. Detection followed by identification of the functional groups of MEL in a frequency range of 400–4000 cm^−1^ with the aid of the Fourier transform infrared spectroscopy technique FTIR (Shimadzu IRAffinity-1 S, Japan), according to the manufacturer’s instructions.

GC/MS-MS Agilent 7000E Triple Quadrupole Gas Chromatograph/Mass Spectrometer (GC/TQ GC/MS-MS) The model is 7000E, a part of Agilent’s 7000 Series https://www.agilent.com/en/product/gas-chromatography-mass-spectrometry-gc-ms/gc-ms-instruments/7000e-triple-quadrupole-gc-ms. (GC/MS-MS having an Elite-5MS with a capillary column (30 × 0.25 μm ID x 0.25 μm df). The ionizing energy was flowed at a constant rate of 1 ml/min with an injection volume of 2 µl (split ratio of 30:1); the injector temperature was 250º C; the isothermal oven temperature was set to 110º C for 2 min increased by 10º C/min reaching 200º C, and terminating by isothermal 9 min at 280º C. Mass spectra were considered at 70 eV. Every scan interval continued for half a second. Fragments were (45-450Da). The run continued for 36 min.

Turbomass software (TurboMassvVer6.1.0 6.1) https://turbomass-ver6-1-0.software.informer.com/versions/ was utilized to operate mass spectra along with chromatograms. The spectral search was performed by using the National Institute of Standards and Technology database (NIST MS Search Program v.2.3). The name, molecular weight, and structures of all compounds were ascertained^57^.

### Assessment of in vitro radical scavenging and antioxidant effect by DPPH (2,2-Diphenyl-1-picrylhydrazyl) assay

The principle of the DPPH assay is to measure the extent of scavenging of the DPPH radicals by MEL. A hydrogen atom is donated by the antioxidants to the nitrogen in the DPPH radical, which has an unpaired electron. This reduced form of DPPH radical and the resultant hydrazine (DPPH-H) lets the violet color disappear considerably to pale yellow. The intensity is measured calorimetrically by spectroscopy^58^. The analysis conditions were 24.3 °C, and the RH% was 47%. The intensity of the discoloration was estimated calorimetrically using a spectrophotometer at 517 nm. Absolute methanol was blank, and the DPPH solution alone was the control. Measurements were done in triplicate, and the average values were considered^59^, and any decrease in the MEL’s absorption in comparison to the control was considered^60^.

The following formula was used to compute the percentage of antioxidants or RSA% % of antioxidant activity= [(Ac − At) ÷ Ac] × 100.

where: Ac: Control reaction absorbance; At: Testing specimen absorbance.

### Experimental animals

Forty albino *Wistar* male rats were fetched from the Holding Company for Biological Products and Vaccines (VACSERA). Rats of seven weeks old weighing 150 ± 10 g. Rats were housed in the Department of Pharmacology, Veterinary Medicine, Cairo University, Egypt, under hygienic laboratory standards. Rats were acclimatized for 14 days in clean bedded cages adjusted to appropriate conditions with free access to water and balanced feed. The protocol complied with the National Research Council’s guidelines. The Institutional Animal Care and Use Committee (IACUC) approval reference number Vet CU 25,122,023,871. Ethical attempts were implemented to treat the experimental animals with empathy and compassion. In addition, all procedures for animal experiments described in this study were performed by the care and use of laboratory animals and ARRIVE guidelines.

### Experimental design

Male *Wistar* albino rats were randomly divided into four groups, with each group comprising ten rats, as follows:

Group 1 (Control) received 0.5 ml/day of vehicle by oral gavage.

Group 2 received RIF orally (gavage) at a dose of 100 mg/kg b.wt^61^.

Group 3 received MEL (10 mg/kg b.wt.)^62^.

Group 4 received RIF with MEL at the same previous doses and regimes.

The treatment was carried out daily over 21 days (3 weeks) in all groups.

### Sampling

Blood specimens were drawn from the retro-orbital plexus by heparinized micro-hematocrit capillary tubes, one week following the inception (just pilot samples to check the start of toxicity) and a later blood sampling at the cessation of the study after the last administration (used for assessment). The last samples were subsequently fractioned for ten minutes with a centrifugation force of 3000 rpm. The pipetted plasma was analyzed for biochemical and lipid profile analyses.

Rats will be euthanized by cervical dislocation under thiopental anesthesia. During the necropsy, the dissected liver weights were determined after washing with physiological saline. Two groups of liver specimens were formed. The initial specimens were fixed in a 10% buffered neutral formalin solution for histological examination. Tissue homogenates were prepared from the latter samples for the oxidative stress and lipid peroxidation assays defined below.

### Biochemical analysis

Plasma was frozen at −20 °C for subsequent analysis. Biodiagnostic commercial kits, for diagnostic and research reagents in Egypt, were utilized for assessing albumin’s concentration^63^ aspartate aminotransferase (AST), and alanine aminotransferase (ALT)^64^, as well as alkaline phosphatase (ALP)^65^ levels in respective aspects.

### Lipid profile assay

Measurement of plasma cholesterol in compliance with Abel et al.^66^, in addition to triglycerides assessment as indicated. High–density lipoprotein cholesterol (HDL-C) level was measured using the method described by Assmann et al.^65^, and low–density lipoprotein cholesterol (LDL-C) was formalized mathematically^66^.

### Hepatic oxidative stress and lipid peroxidation markers

Hepatic tissue homogenates were prepared: 0.5 g hepatic tissue was washed initially in 0.9% normal saline and made homogenate in ice-cold phosphate buffer (pH 7.4). The homogenate was centrifuged at 6000×g for 10 min at 4 °C. Then the supernatant was aspirated and stored at − 80 °C for assays. Biodiagnostic kits, for diagnostic and research reagents in Egypt, were utilized to estimate hepatic superoxide dismutase (SOD) colorimetrically^69^, malondialdehyde (MDA)^70^, as well as glutathione peroxidase (GPX)^71^.

### Histological examination

Dissection of the liver from each rat was done, followed by specimen preservation in a 10% buffered neutral formalin solution for fixation. This was followed by dehydration with ascending alcohol concentrations. They were then cleared using xylene. Routine processing and sectioning at 44-µm-thick sections stained with Hematoxylin and Eosin (H&E)^72^. A conventional microscope (Olympus, Tokyo, Japan) was utilized at different magnification powers. The histopathological changes were recorded by using a scoring system (0–5)^26^.

### Molecular-docking analysis

Virtual screening techniques were used to gain insight into the hepatoprotective mechanism of MEL. The interrelation between MEL, cinnamic acid, and CYP3A4 was considered. Homo sapiens CYP3A4 bound to an inhibitor, metyrapone ID: 6MA6, was chosen from the protein data bank (https://www.rcsb.org) with no sequence mutation and a resolution of 2.18 Å^73^. Biovia Discovery Studio Visualizer was utilized to prepare the receptor by discarding the co-crystallized ligands and other concomitant solvents^74^. MEL’s ID: 896 and cinnamic acid’s ID: 444,539 were procured from the National Center for Biotechnology Information web server, NCBI, USA: PubChem database (https://pubchem.ncbi.nlm.nih.gov/). Avogadro Molecular Editor and Visualization Tool version 1.2.0 (https://avogadro.cc) was used for optimization and energy minimization using various algorithms^75^. Docking was processed, and Gibbs free energy (ΔG) was estimated by the virtual screening software for computational drug discovery. The PyRx–Virtual Screening Tool version 0.8 (https://pyrx.sourceforge.io) was utilized for virtual screening and molecular docking purposes^76^. BIOVIA Discovery Studio Visualizer version: 24.1.0 (https://discover.3ds.com/discovery-studio-visualizer-download) and UCSF ChimeraX–Next-generation molecular visualization program from UCSF Version: 1.7 (https://www.cgl.ucsf.edu/chimerax) were used for 2D and 3D interaction visualizations, such as plots, and other aspects of the graphical interface, as well as analyzing hydrogen bonds^76^.

### Statistical analysis

Raw data were collected and generated with the aid of one-way variance analysis (ANOVA) through SPSS version 14 (SPSS, Chicago, IL, USA). Duncan’s Multiple Range Test determined the mean difference. Remarking that any p-value below 0.05 is considered statistically significant. The Shapiro-Wilk W test examined normality for the entire dataset.

The changes in different groups were statistically analyzed with SPSS-14 software. One-way variance analysis and LSD post-test were used to evaluate significant differences between groups.

## Supplementary Information

Below is the link to the electronic supplementary material.

Supplementary Material 1

## Data Availability

All data supporting the findings of this study are available within the paper and its supplementary file.

## References

[1] Zhang, G. et al. Hesperidin alleviates oxidative stress and upregulates the multidrug resistance protein 2 in Isoniazid and Rifampicin-Induced liver injury in rats. *J. Biochem. Mol. Toxicol.* **30**, 342–349 (2016). 27017938 10.1002/jbt.21799

[2] Yang, J. et al. Effects of medium- and long-chain fatty acids on acetaminophen- or rifampicin-induced hepatocellular injury. *Food Sci. Nutr.* **8**, 3590–3601 (2020). 32724621 10.1002/fsn3.1641 PMC7382196

[3] Dirlikov, E., Raviglione, M. & Scano, F. Global tuberculosis control: toward the 2015 targets and beyond. *Ann. Intern. Med.* **163**, 52–58 (2015). 25915859 10.7326/M14-2210

[4] Combrink, M. & Loots, D. T. & du preez, I. Metabolomics describes previously unknown toxicity mechanisms of Isoniazid and rifampicin. *Toxicol. Lett.* **322**, 104–110 (2020). 31981687 10.1016/j.toxlet.2020.01.018

[5] Soni, H., Gautam, D. V. K., Sharma, S. & Malik, J. K. Rifampicin as potent inhibitor of COVID-19 main protease: In-Silico Docking approach. *Saudi J. Med. Pharm. Sci.* **6**, 588–593 (2020).

[6] Edwards, B. D., Mah, H., Sabur, N. F. & Brode, S. K. Hepatotoxicity and tuberculosis treatment outcomes in chronic liver disease. *J. Assoc. Med. Microbiol. Infect. Dis. Can.* **8**, 65–74 (2023). 10.3138/jammi-2022-0029 PMC10052910 37008589

[7] Baskaran, U. L. & Sabina, E. P. Clinical and experimental research in antituberculosis drug-induced hepatotoxicity: a review. *J. Integr. Med.* **15**, 27–36 (2017). 28088257 10.1016/S2095-4964(17)60319-4

[8] Yuan, L., Na, H. & Li, W. An excerpt of Drug-induced liver injury: Asia Pacific association of study of liver consensus guidelines (2021). *J. Clin. Hepatol.* **37**, 1291–1294 (2021). 10.1007/s12072-021-10144-3 33641080

[9] Hou, W., Nsengimana, B., Yan, C., Nashan, B. & Han, S. Involvement of Endoplasmic reticulum stress in rifampicin-induced liver injury. *Front. Pharmacol.* **13**, 1–8 (2022). 10.3389/fphar.2022.1022809 PMC9630567 36339603

[10] Reiter, R. J., Tan, D., Sainz, R. M., Mayo, J. C. & Lopez-Burillo Melatonin: reducing the toxicity and increasing the efficacy of drugs. *J. Pharm. Pharmacol.* **54**, 1299–1321 (2010). 10.1211/002235702760345374 12396291

[11] Dorranipour, D., Pourjafari, F., Malekpour-Afshar, R., Basiri, M. & Hosseini, M. Assessment of melatonin’s therapeutic effectiveness against hepatic steatosis induced by a high-carbohydrate high-fat diet in rats. *Naunyn Schmiedebergs Arch. Pharmacol.* **397**, 2971–2985 (2024). 37864588 10.1007/s00210-023-02784-z

[12] Zhang, J. J. et al. Effects of melatonin on liver injuries and diseases. *Int. J. Mol. Sci.* **18**, 673 (2017). 28333073 10.3390/ijms18040673 PMC5412268

[13] Shafiq-ur-Rehman. Evaluation of malondialdehyde as an index of Chlorpyriphos insecticide exposure in *Apis mellifera*: ameliorating role of melatonin and α-tocopherol against oxidative stress. *Toxicol. Environ. Chem.* **91**, 1135–1148 (2009).

[14] Reiter, R. J., Tan, D. X. & Galano, A. Melatonin: exceeding expectations. *Physiology* **29**, 325–333 (2014). 25180262 10.1152/physiol.00011.2014

[15] Mi, L. & Kuang, H. Melatonin regulates cisplatin resistance and glucose metabolism through Hippo signaling in hepatocellular carcinoma cells. *Cancer Manag Res.* **12**, 1863–1874 (2020). 32210629 10.2147/CMAR.S230466 PMC7075351

[16] Reiter, R. J. et al. Melatonin as an antioxidant: underpromises but over-delivers. *J. Pineal Res.* 253–278. 10.1111/jpi.12360 (2016). 10.1111/jpi.12360 27500468

[17] Sinha, P. et al. Undernutrition and tuberculosis: public health implications. *J. Infect. Dis.* **219**, 1356–1363 (2019). 30476125 10.1093/infdis/jiy675 PMC6941617

[18] Awodele, O., Akintonwa, A., Osunkalu, V. O. & Coker, H. A. B. Modulatory activity of antioxidants against the toxicity of rifampicin in vivo. *Rev. Inst. Med. Trop. Sao Paulo*. **52**, 43–46 (2010). 20305954 10.1590/s0036-46652010000100007

[19] Reiter, R. J., Paredes, S. D., Manchester, L. C. & Tan, D. X. Reducing oxidative/nitrosative stress: a newly-discovered genre for melatonin. *Crit. Rev. Biochem. Mol. Biol.* **44**, 175–200 (2009). 19635037 10.1080/10409230903044914

[20] Gu, C. et al. Melatonin rescues the mitochondrial function of bone marrow-derived mesenchymal stem cells and improves the repair of osteoporotic bone defects in ovariectomized rats. *J Pineal Res* **76**(1), e12924 (2024). 10.1111/jpi.12924 37941528

[21] Ismaili, L., Romero, A., Carmo Carreiras, M. & Marco-Contelles, J. do in *Design of Hybrid Molecules for Drug Development* 5–46 (2017). 10.1016/B978-0-08-101011-2.00002-7

[22] León, R., Garcia, A. G. & Marco-Contelles, J. Recent advances in the multitarget-directed ligands approach for the treatment of alzheimer’s disease. *Med. Res. Rev.* **33**, 139–189 (2013). 21793014 10.1002/med.20248

[23] Buendia, I. et al. New melatonin-cinnamate hybrids as multi-target drugs for neurodegenerative diseases: Nrf2-induction, antioxidant effect, and neuroprotection. *Future Med. Chem.* **7**, 1961–1969 (2015). 26496465 10.4155/fmc.15.99

[24] Florio, G. M., Christie, R. A., Jordan, K. D. & Zwier, T. S. Conformational preferences of jet-cooled melatonin: probing trans- and cis-amide regions of the potential energy surface. *J. Am. Chem. Soc.* **124**, 10236–10247 (2002). 12188688 10.1021/ja0265916

[25] Asad-Ullah, M. A. et al. Effect of ultraviolet-C radiation and melatonin stress on biosynthesis of antioxidant and antidiabetic metabolites produced in vitro callus cultures of *Lepidium sativum* L. *Int J. Mol. Sci* **20**(7), 1–9 (2019). 10.3390/ijms20071787 PMC6479895 30978911

[26] Maiti, S., Parua, S., Nandi, D. K., Mondal, K. C. & Samanta, S. Hepatotoxic effect of rifampicin as an Anti-Tuberculosis drug on male albino rats. *J. Drug Deliv Ther.* **9**, 26–32 (2019).

[27] Kim, J. H. et al. Mechanism investigation of rifampicin-induced liver injury using comparative toxicoproteomics in mice. *Int J. Mol. Sci* **18**, 1–13 (2017). 10.3390/ijms18071417 PMC5535909 28671602

[28] Rana, S. V., Pal, R., Vaiphie, K. & Singh, K. Effect of different oral doses of isoniazid-rifampicin in rats. *Mol. Cell. Biochem.* **289**, 39–47 (2006). 16583132 10.1007/s11010-006-9145-3

[29] Yuhas, Y., Berent, E. & Ashkenazi, S. Effect of Rifampin on production of inflammatory mediators in HepG2 liver epithelial cells. *Antimicrob. Agents Chemother.* **55**, 5541–5546 (2011). 21930886 10.1128/AAC.05149-11 PMC3232822

[30] Mohamed, R. S., Fouda, K. & Akl, E. M. Hepatorenal protective effect of flaxseed protein isolate incorporated in lemon juice against lead toxicity in rats. *Toxicol. Rep.* **7**, 30–35 (2020). 31890606 10.1016/j.toxrep.2019.12.001 PMC6926353

[31] Chawla, S. L., Yadav, R., Shah, D. & Rao, M. V. Protective action of melatonin against fluoride-induced hepatotoxicity in adult female mice. *Fluoride* **41**, 44–51 (2008).

[32] Huang, J. H. et al. Rifampicin-induced hepatic lipid accumulation: association with up-regulation of peroxisome proliferator-activated receptor ã in mouse liver. *PLoS One* **11**(11), e0165787 (2016). 10.1371/journal.pone.0165787 PMC5091861 27806127

[33] Ahmed, N. et al. Effects of Anti-Tuberculosis drugs on lipid profile in pulmonary tuberculosis patients. *J. Dow Univ. Heal Sci.* **16**, 78–82 (2022).

[34] Abdel-Wahab, W. M. AlCl3-induced toxicity and oxidative stress in the liver of male rats: protection by melatonin. *Life Sci. J.* **9**, 1173–1182 (2012).

[35] Antolín, I. et al. Neurohormone melatonin prevents cell damage: effect on gene expression for antioxidant enzymes. *FASEB J.* **10**, 882–890 (1996). 8666165 10.1096/fasebj.10.8.8666165

[36] Ding, K. et al. Melatonin stimulates antioxidant enzymes and reduces oxidative stress in experimental traumatic brain injury: the Nrf2-ARE signaling pathway as a potential mechanism. *Free Radic Biol. Med.* **73**, 1–11 (2014). 24810171 10.1016/j.freeradbiomed.2014.04.031

[37] López-Burillo, S. et al. Melatonin, xanthurenic acid, resveratrol, EGCG, vitamin C, and α-lipoic acid differentially reduce oxidative DNA damage induced by Fenton reagents: A study of their individual and synergistic actions. *J. Pineal Res.* **34**, 269–277 (2003). 12662349 10.1034/j.1600-079x.2003.00041.x

[38] Melchiorri, D. et al. Potent protective effect of melatonin on in vivo paraquat-induced oxidative damage in rats. *Life Sci.* **56**, 83–89 (1994). 10.1016/0024-3205(94)00417-q 7823762

[39] López-Burillo, S. et al. Melatonin and its derivatives, Cyclic 3-hydroxymelatonin, N1-acetyl-N2-formyl-5-methoxykynuramine, and 6-methoxymelatonin, reduce oxidative DNA damage induced by Fenton reagents. *J. Pineal Res.* **34**, 178–184 (2003).

[40] Nuñez, P. & Zapico, S. C. in *Free Radicals and Health* 75–98 (2016).

[41] Reina, M. & Martínez, A. A new free radical scavenging cascade involving melatonin and three of its metabolites (3OHM, AFMK, and AMK). *Comput. Theor. Chem.* **1123**, 111–118 (2018).

[42] Ramappa, V. & Aithal, G. P. Hepatotoxicity related to Anti-tuberculosis drugs: mechanisms and management. *J. Clin. Experimental Hepatol.* **3**, 37–49 (2013). 10.1016/j.jceh.2012.12.001 PMC3940184 25755470

[43] Yan, J. & Xie, W. A brief history of the discovery of PXR and CAR as xenobiotic receptors. *Acta Pharm. Sinica B*. **6**, 450–452 (2016). 10.1016/j.apsb.2016.06.011 PMC5045536 27709013

[44] Sennesael, A. L. et al. The impact of strong inducers on direct oral anticoagulant levels. *Am. J. Med.* **134**, 1295–1299 (2021). 34181907 10.1016/j.amjmed.2021.06.003

[45] Panayiotakopoulos, G. D. & Papadimitriou, D. T. Rifampicin for COVID-19. *World J. Virol.* **11**, 90–97 (2022). 35433334 10.5501/wjv.v11.i2.90 PMC8966591

[46] Baneyx, G., Parrott, N., Meille, C., Iliadis, A. & Lavé, T. Physiologically based Pharmacokinetic modeling of CYP3A4 induction by rifampicin in human: influence of time between substrate and inducer administration. *Eur. J. Pharm. Sci.* **56**, 1–15 (2014). 24530864 10.1016/j.ejps.2014.02.002

[47] Chattopadhyay, N. et al. CYP3A4-mediated effects of rifampicin on the pharmacokinetics of Vilaprisan and its UGT1A1-mediated effects on bilirubin glucuronidation in humans. *Br. J. Clin. Pharmacol.* **84**, 2857–2866 (2018). 30171692 10.1111/bcp.13750 PMC6256003

[48] Veith, A. & Moorthy, B. Role of cytochrome P450s in the generation and metabolism of reactive oxygen species. *Curr. Opin. Toxicol.* **7**, 44–51 (2018). 29527583 10.1016/j.cotox.2017.10.003 PMC5841237

[49] Kivrane, A. et al. Exploring variability in rifampicin plasma exposure and development of Anti-Tuberculosis Drug-Induced liver injury among patients with pulmonary tuberculosis from the Pharmacogenetic perspective. *Pharmaceutics* **16**, 388 (2024). 38543282 10.3390/pharmaceutics16030388 PMC10974048

[50] Eugenia, M. et al. Chemico-Biological interactions: melatonin protects the cytochrome P450 system through a novel antioxidant mechanism. **185**, 208–214 (2010). 10.1016/j.cbi.2010.03.020 20302852

[51] Nishimura, J. et al. Antioxidant, enzymatically modified isoquercitrin, or melatonin supplementation reduces oxidative stress-mediated hepatocellular tumor promotion of oxfendazole in rats. *Arch. Toxicol.* **84**, 143–153 (2010). 20033131 10.1007/s00204-009-0497-9

[52] He, L. et al. Synthesis of melatonin derivatives and the neuroprotective effects on parkinson’s disease models of caenorhabditis elegans. *Front Chem* **10**, 1–10 (2022). 10.3389/fchem.2022.918116 PMC9213837 35755259

[53] Esmaeili, S. et al. Exogenous melatonin induces phenolic compounds production in Linum album cells by altering nitric oxide and Salicylic acid. *Sci Rep* **13**, 1–12 (2023). 10.1038/s41598-023-30954-9 PMC10011386 36914704

[54] Park, J. H. et al. Protective effects of Melittin on tumor necrosis factor-α-induced hepatic damage through suppression of apoptotic pathway and nuclear factor-kappa B activation. *Exp. Biol. Med.* **239**, 1705–1714 (2014). 10.1177/1535370214533880 24872433

[55] Sewerynek, E. et al. Melatonin administration prevents lipopolysaccharide-induced oxidative damage in phenobarbital‐treated animals. *J. Cell. Biochem.* **58**, 436–444 (1995). 7593265 10.1002/jcb.240580406

[56] Oesch, F., Arand, M., Strolin Benedetti, M., Castelli, M. G. & Dostert, P. Inducing properties of rifampicin and Rifabutin for selected enzyme activities of the cytochrome P-450 and UDP-glucuronosyltransferase superfamilies in female rat liver. *J. Antimicrob. Chemother.* **37**, 1111–1119 (1996). 8836814 10.1093/jac/37.6.1111

[57] Ezhilan, B. P. & Neelamegam, R. GC-MS analysis of phytocomponents in the ethanol extract of polygonum Chinense L. *Pharmacognosy Res.* **4**, 11–14 (2012). 22224055 10.4103/0974-8490.91028 PMC3250032

[58] Gulcin, İ. & Alwasel, S. H. *DPPH Radical Scavenging Assay. Processes* **11**(8), 1–20 (2023).

[59] Ansari, A. Q., Ahmed, S. A., Waheed, M. A. & Juned, S. Extraction and determination of antioxidant activity of Withania somnifera Dunal. *Eur. J. Exp. Biol.* **3**, 502–507 (2013).

[60] Naji, K. M., Al-Khatib, B. Y., Al-Haj, N. S. & D’souza, M. R. Hepatoprotective activity of Melittin on isoniazid- and rifampicin-induced liver injuries in male albino rats. *BMC Pharmacol. Toxicol.* **22**, 39 (2021). 34217369 10.1186/s40360-021-00507-9 PMC8254969

[61] Jahovic, N., Çevik, H., Şehirli, A. Ö., Yeǧen, B. Ç. & Şener, G. Melatonin prevents methotrexate-induced hepatorenal oxidative injury in rats. *J. Pineal Res.* **34**, 282–287 (2003). 12662351

[62] Høstmark, A. T., Tomten, S. E. & Berg, J. E. Serum albumin and blood pressure: A population-based, cross-sectional study. *J. Hypertens.* **23**, 725–730 (2005). 15775775 10.1097/01.hjh.0000163139.44094.1d

[63] Rani, J. & Raju, D. Estimation of serum glutamic oxaloacetic transaminase, serum glutamic-pyruvic transaminase, gamma-glutamyl transferase, and cholesterol levels in prolonged (30 years) daily consumption of coffee in people. *Int. J. Res. Med. Sci.* 1564–1573. 10.18203/2320-6012.ijrms20161228 (2016).

[64] Kind, P. R. & King, E. J. Estimation of plasma phosphatase by determination of hydrolyzed phenol with amino-antipyrine. *J. Clin. Pathol.* **7**, 322–326 (1954). 13286357 10.1136/jcp.7.4.322 PMC1023845

[65] Abel, L. L., Levy, B. B., Brodie, B. B. & Kendall, F. E. A simplified method for the Estimation of total cholesterol in serum and demonstration of its specificity. *J. Biol. Chem.* **195**, 357–366 (1952). 14938387

[66] Bucolo, G. & David, H. Quantitative determination of serum triglycerides by the use of enzymes. *Clin. Chem.* **19**, 476–482 (1973). 4703655

[67] Assmann, G., Schriewer, H., Schmitz, G. & Hagele, E. O. Quantification of high-density-lipoprotein cholesterol by precipitation with phosphotungstic acid/MgCl2. *Clin. Chem.* **29**, 2026–2030 (1983). 6640896

[68] Durak, I., Yurtarslanl, Z., Canbolat, O. & Akyol, Ö. A methodological approach to superoxide dismutase (SOD) activity assay based on Inhibition of nitroblue tetrazolium (NBT) reduction. *Clin. Chim. Acta*. **214**, 103–104 (1993). 8453769 10.1016/0009-8981(93)90307-p

[69] Ohkawa, H., Ohishi, N. & Yagi, K. Assay for lipid peroxides in animal tissues by thiobarbituric acid reaction. *Anal. Biochem.* **95**, 351–358 (1979). 36810 10.1016/0003-2697(79)90738-3

[70] Paglia, D. E. & Valentine, W. N. Studies on the quantitative and qualitative characterization of erythrocyte glutathione peroxidase. *J. Lab. Clin. Med.* **70**, 158–169 (1967). 6066618

[71] Suvarna, S. K., Layton, C. & Bancroft, J. D. *Bancroft’s Theory and Practice of Histological Techniques, Eighth Edition* (2018). 10.1016/C2015-0-00143-5

[72] Hiraizumi, M. et al. Transport and Inhibition mechanism of the human SGLT2-MAP17 glucose transporter. *Nat. Struct. Mol. Biol.*10.1038/s41594-023-01134-0 (2023). 38057552 10.1038/s41594-023-01134-0 PMC10803289

[73] Dassault Systèmes. Biovia Discovery Studio provides comprehensive modeling and simulations for life sciences. Dassault Systèmes. (2023). Available at: https://discover.3ds.com/discovery-studio-visualizer-download

[74] López, R. Capillary surfaces with free boundary in a wedge. *Adv. Math. (N Y)*. **262**, 476–483 (2014).

[75] Dallakyan, S. & Olson, A. Participation in global governance: coordinating ‘the voices of those most affected by food insecurity’. *Glob Food Secur. Gov.* **1263**, 1–11 (2015).

[76] Meng, E. C. et al. UCSF chimerax: tools for structure Building and analysis. *Protein Sci.* **32**, 1–13 (2023). 10.1002/pro.4792 PMC10588335 37774136

