# Supplementary material for: Combined in vivo and silico assessment of melatonin’s protective effects on rifampicin-induced liver damage in rats
Source: Sci Rep. 2025 Aug 24;15:31104. doi: 10.1038/s41598-025-16453-z (PMC12375711; doi:10.1038/s41598-025-16453-z)
Supplement: Supplementary file 1 — Supplementary Material 1 [file 41598_2025_16453_MOESM1_ESM.docx]

**Supplementary Table 1**: Effect of melatonin on hepatic oxidative stress and lipid peroxidation markers in Wistar male rats exposed to oral dosing of rifampicin for 21 days

| **Estimated parameter** | **Control** | **RIF** | **MEL** | **RIF+MEL** |
| --- | --- | --- | --- | --- |
| **SOD (U/g)** | 50.30^a^±2.37 | 13.77^d^±1.60 | 57.40^a^±2.46 | 27.17^b^±0.98 |
| **MDA (Nmol/g)** | 8.77^a^±0.80 | 42.00^c^±1.76 | 6.43^a^±0.97 | 24.33^b^±1.12 |
| **GPX (U/g)** | 31.13^a^±1.10 | 7.43^c^±0.80 | 35.40^a^±1.59 | 20.50^b^±0.74 |

SOD: Hepatic superoxide dismutase, MDA: malondialdehyde, and GPX: glutathione peroxidase. Means within the same row carrying different superscripts are significantly different at *p* < 0.05. The values shown are means ± SE. n = 10.

| **List of abbreviations** | |
| --- | --- |
| MEL | Melatonin |
| RIF | Rifampicin |
| TB | Tuberculosis |
| FTIR | Fourier transform infrared spectroscopy |
| GC–MS | Gas chromatography-mass spectrometry |
| DPPH | 2,2-diphenyl-1-picrylhydrazyl radical scavenging assay |
| ALP | Alkaline phosphatase |
| ALT | Alanine aminotransferase |
| AST | Aspartate aminotransferase |
| TG | Triglycerides |
| TC | Total cholesterol |
| LDL | Low-density lipoprotein |
| HDL | High-density lipoprotein |
| GPX | Glutathione peroxidase |
| MDA | Malondialdehyde |
| SOD | Superoxide dismutase |
| NIST | National Institute of Standard and Technology database |
| CAS NO. | Chemical abstracts service number |
| CMC | Carboxymethyl cellulose |
| IACUC | The Institutional Animal Care and Use Committee |
| HDL-C | High–density lipoprotein cholesterol |
| LDL-C | Low–density lipoprotein cholesterol |
| H&E | Hematoxylin and Eosin |
| RLD | Reference listed drug |
| FDA | Food and Drug Administration |
| NCBI | National Center for Biotechnology Information web server |
| UCSF | University of California San Francisco Parnassus Campus |
| RSA | The free radical scavenging activity |
| RMSD | Root mean square deviation |
| 2D | Two dimensional |
| 3D  CYP450 | Three dimensional  cytochrome P450 |
| CYP3A4 | cytochrome P4503A4 |
